# Research areas and trends in family-centered care in the 21st century: a bibliometric review

**DOI:** 10.3389/fmed.2024.1401577

**Published:** 2024-06-11

**Authors:** Mojca Hriberšek, Fabian Eibensteiner, Nils Bukowski, Andy Wai Kan Yeung, Atanas G. Atanasov, Eva Schaden

**Affiliations:** ^1^Ludwig Boltzmann Institute Digital Health and Patient Safety (LBG), Vienna, Austria; ^2^Medical University of Vienna, Vienna, Austria; ^3^Clinical Department of Pediatric Nephrology and Gastroenterology, University Clinic for Pediatrics and Adolescent Medicine, Medical University of Vienna, Vienna, Austria; ^4^Department of Anaesthesia, Intensive Care Medicine and Pain Medicine, Medical University of Vienna, Vienna, Austria; ^5^Faculty of Dentistry, The University of Hong Kong, Hong Kong SAR, China; ^6^Institute of Genetics and Animal Biotechnology, Polish Academy of Sciences, Magdalenka, Poland

**Keywords:** neonatal care, pediatrics, adult medicine, intensive care, palliative care, end-of-life care, family involvement, digitalisation

## Abstract

**Introduction:**

Family-centered care (FCC) is a model of care provision that sees a patient’s loved ones as essential partners to the health care team and positively influences the psychological safety of patients and loved ones.

**Objectives:**

This review aims to present an overview of impactful publications, authors, institutions, journals, countries, fields of application and trends of FCC in the 21^st^ century as well as suggestions on further research.

**Methods:**

The Web of Science Database was searched for publications on FCC between January 2000 and Dezember 2023. After screening for duplicates, VOS Viewer and CiteSpace were used to analyze and visualize the data.

**Results:**

Scientific interest in FCC has grown and resulted in the scientific output of 4,836 publications originating from 103 different countries. Based on the frequent author keywords, FCC was of greatest interest in neonatology and pediatrics, nursing, critical and intensive care, end-of-life and palliative care, and patient-related outcomes. The recent research hotspots are “patient engagement,” “qualitative study,” and “health literacy.”

**Conclusion:**

FCC has gained recognition and spread from the pediatric to the adult palliative, intensive, end-of-life and geriatric care settings. This is a very reassuring development since adults, especially when older, want and need the assistance of their social support systems. Recent research directions include the involvement of patients in the development of FCC strategies, health literacy interventions and the uptake of telemedicine solutions.

## 1 Introduction

Two major systems support patients on their healthcare journey: the healthcare system and their social circle (family, friends, caregivers, or other loved ones). Family-centered care (FCC) defines a patient’s loved ones as a source of strength and support, and sees them as inseparable unit and/or equal partners to patients and healthcare professionals (HCPs) in the treatment and management of disease (1, 2). They aim at building a partnership between HCPs and the patients and their social circle and together with shared decision making (SDM), FCC interventions are increasingly being recognized and implemented to improve the quality of healthcare (3, 4). While there is no official definition of FCC, a scoping review from 2019 identified the following key components: (1) collaboration between family members and healthcare providers, (2) consideration of family contexts, (3) education for patients, families, and HCPs, and (4) dedicated policies and procedures (5). Furthermore, the COVID-19 pandemic and associated hospital visitation restrictions have served as a reminder of the importance of the presence of loved ones in the patient’s healthcare journey (6, 7).

Overall, FCC promotes a respectful partnership between the provider and receiver of care (patient and their closest social support circle), supports a culture of patient safety as well as psychological safety (1, 8) and furthermore helps build health-care and management competences that result in community empowerment. The ability to speak up regarding your thoughts and concerns without interpersonal risk is essential in an environment such as healthcare. Independent of care setting, family members actively engaged in the care of their loved ones can serve as additional eyes and ears, helping to identify potential safety concerns or issues and ensuring timely interventions (9). Moreover, healthcare providers can gain valuable insights and perspectives that can contribute to safer and more effective care (3). Thereby, healthcare systems that embrace family- and patient-centeredness and mutual acknowledgement support patient participation in safety practices and increase psychological as well as patient safety (10).

Hence, to analyze the whole literature of FCC regarding their origin, content and impact, the bibliometric review was chosen as the appropriate method. To date, only systematic and scoping reviews summarizing the growing body of evidence on FCC have been published and while these review methods focus on a specific research question and implementations and their results, they do not provide a comprehensive overview of the existing literature.

The purpose of this paper is, firstly, to highlight impactful publications, authors, journals, institutions, and countries on FCC, secondly, to provide an overview of the medical fields in which FCC is applied, thirdly, to provide insight into past and current trends within FCC and, finally, to offer suggestions for further research.

## 2 Methods

### 2.1 Design

Bibliometric review is a method used to depict and analyze cumulative scientific knowledge in an area of interest. It can be used to (1) gain an overview and uncover new research trends, co-working patterns among single authors, organizations or countries, and define impactful research works, (2) identify knowledge gaps, (3) derive novel ideas for investigation, and (4) position new research to contribute to the field (11). Bibliometric analyses have already been used to give a literature overview of 21st century developments in topics such as SDM (12) and palliative care research (13). The review was conducted according to the Prisma-ScR extension framework, which is the closest to the methodology of a bibliometric review, as no framework for bibliometric reviews exists.

### 2.2 Search methods

Data for this bibliometric review was extracted from Web of Science on October 10, 2023, at the Medical University of Vienna, Vienna, Austria. To identify FCC developments in the 21st century, the time span was set from January 1, 2000, to December 31, 2022. Due to the different spellings in British and American English (family-centered/family-centered), the following search query was used “TS = (“family-centered*” or “family-centered*”) and TS = (“care”).” Titles, abstracts, and keywords (both author keywords and KeyWords Plus (14) were scanned, ensuring that not only publications explicitly mentioning FCC but also those on topic of FCC were taken into account.

### 2.3 Inclusion criteria

As this was a comprehensive analysis of the literature in question, all resulting papers were included without exception (Multimedia Appendix 1).

### 2.4 Search outcomes

The identified papers were scanned for duplicates and since none were found, all identified papers were submitted to analysis.

### 2.5 Quality appraisal

Since the bibliometric review aims to analyze the whole body of literature on FCC in the 21^st^ century, the focus of this analysis is on the topics, types and contributors of the research and not on the quality of the work.

### 2.6 Data abstraction

The identified papers were exported in full record with cited references from Web of Science. The following data were used for analysis: author, institution, country, year of publication, publishing journal, citation count and author keywords.

### 2.7 Synthesis

Data analysis and visualizations were conducted using R software (15) and VOS Viewer (Version 1.6.18) (16). Continuous data was summarized using mean and standard deviation; categorical data was summarized using absolute and relative frequencies. VOS viewer was also used to visualize network maps of the co-authorship connections of organizations and authors, and co-occurrence of author keywords. The maps were generated by constructing a similarity matrix based on association strength, followed by optimization using the majorization algorithm, and finally transformations to ensure consistent solutions (16). The distance-based networks generated visualize items with circles; frequency of occurrence is indicated by node size; strength of relation between items by the length of the edge. When analyzing data, full counting was applied, i.e., all co-authorships and co-occurrences were weighted equally. For authors keywords, a thesaurus file was created to merge the spelling of FCC to “Family-centered care.” Furthermore, thresholds (T) were applied and adapted to select for top contributors. Finally, the VOS Viewer unique variant of modularity-based clustering with a resolution parameter (17) was used to cluster these contributors. Citation bursts – frequency surges of author keywords used in the cited publications – can be used as indicators for research hotspots. They were analyzed using CiteSpace (Version 6.1.R4) (18, 19) under the following parameters: time span (2000–2022), years per slice (1), links (strength: cosine, scope: within slices), term source (Author Keywords), selection criteria (TopN% = 5; max. Items per slice 100). Since.

## 3 Results

### 3.1 Annual output

**Figure 1:**
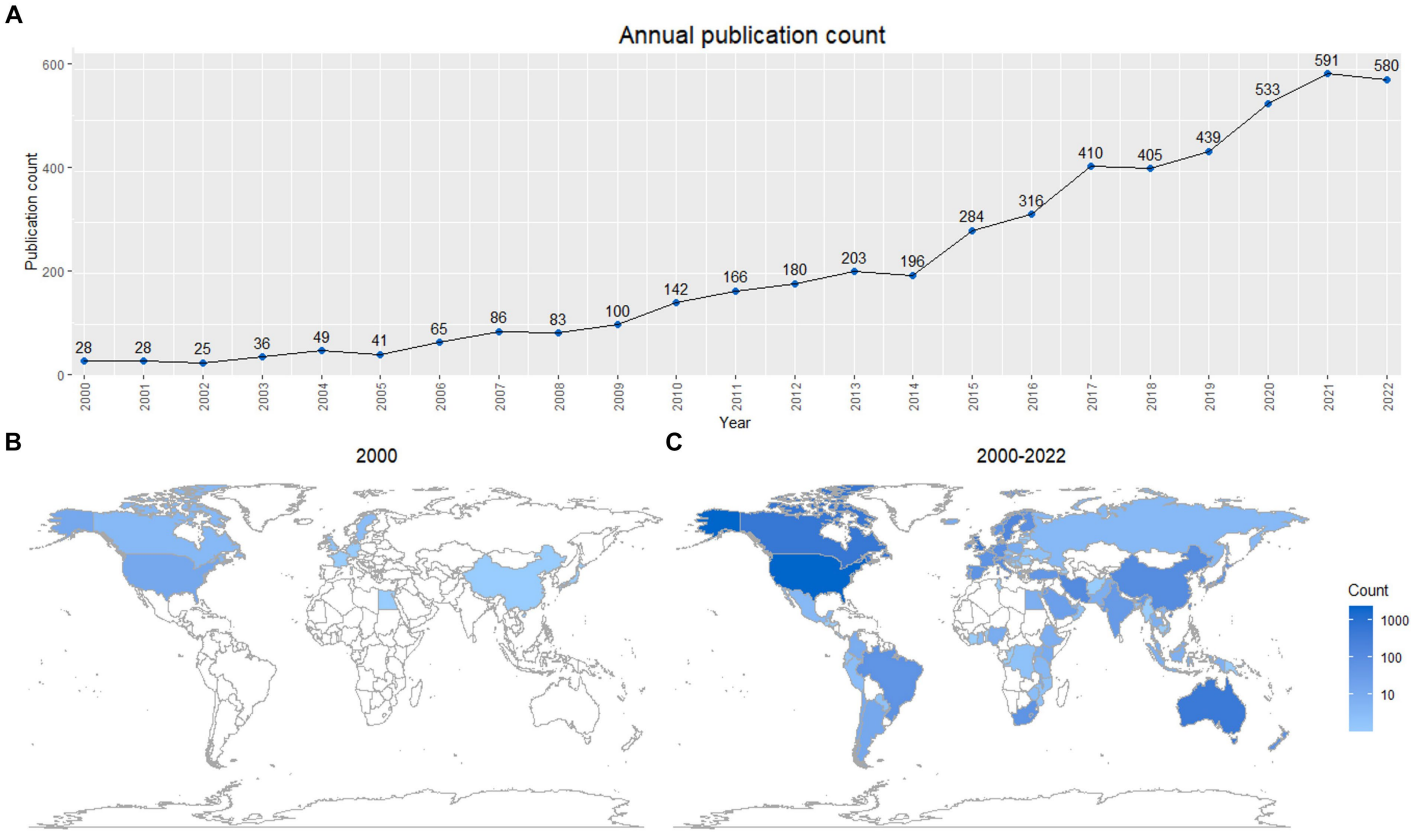
FCC publication rates during the 21st century. **(A)** Annual output of FCC publications in the 21st century. **(B)** Countries publishing on FCC in the year 2000 (9 countries). **(C)** Countries publishing on FCC in the years 2000 to 2022 (103 countries). Scale is logarithmic.

A total of 4,986 FCC-related publications were published and included in the Web of Science core collection between 2000 and 2022. In 2000, only 29 articles were published, while in 2022, this figure had risen to 580 (Figure 1A). Overall, there is a steady increase in the number of published articles over the progress of the 21st century.

### 3.2 Analysis of countries and organizations

Alongside the increase in publication volume, the number of publishing countries also increased. In 2000, 9 countries published FCC-related articles (Figure 1B), while between 2000 to 2022, this figure grew to 103 countries (Figure 1C). The majority of articles were published in the United States (2,438/4663; 52.3%), followed by three other major English-speaking countries: Canada (626/4663; 13.4%), Australia (504/4663; 10.8%) and the United Kingdom (338/4663; 7.2%), amounting to 83.8% (Table 1). Among the top 10 contributing countries, each country contributed a mean of 466.3 ± 680.47 publications. Articles from these countries were cited with an average citations per publication ratio (CPP) of 17.46 ± 5.172.

**Table 1:** Top 10 publishing countries and organizations in the field of family-centered care.

	Publication count	Citation per publication
Country		
USA	2,438	20.40
Canada	626	23.20
Australia	504	15.70
United Kingdom	338	21.46
Sweden	168	21.91
Netherlands	162	20.78
Iran	126	5.52
People’s Republic of China	112	11.81
Italy	98	16.24
Germany	91	17.60
Organization		
University of Toronto	159	22.43
University of Washington	117	31.87
University of Queensland	104	15.47
McMaster University	99	37.17
University of Pennsylvania	91	18.51
Johns Hopkins University	86	28.95
University California - San Francisco	82	65.65
McGill University	82	18.18
University of Calgary	80	20.31
University of Alberta	70	19.53
Author		
Latour, Jos M.	33	18.94
Lyon, Maureen E.	29	22.48
Axelin, Anna	27	29.30
Shields, Linda	27	29.04
Wang, Jichuan	25	21.48
Scarinci, Nerina	25	8.36
Lehtonen, Liisa	23	29.91
Franck, Linda S	21	37.38
Curtis, J. Randall	20	57.55
Davidson, Judy E.	19	53.47

The top 10 publishing organizations contributed a mean of 97.0 ± 24.36 articles related to FCC (Table 1). At 159, the University of Toronto published the most articles, while the highest citation per publication ratio was achieved by University of California – San Francisco with 65.65. The network of top 2% (101/4945) of organizations by publishing volume (at least 21 articles) shows 4 clusters that very largely coincide with the organizations’ host countries (Supplementary Figure S1): The largest cluster contains organizations from the United States; the second largest from Canada; the third largest from Australia, New Zealand and Europe; and the smallest from Iran and United States. Based on this cluster analysis of co-authorship, stronger associations are observable between organizations in the United States and Canada, while Australian and New Zealand organizations are more associated with European organizations.

### 3.3 Analysis of journals

Most papers were published in the journal *Pediatrics,* with a total count of 138 papers (Table 2). The top 10 journals together published 818 articles, accounting for 16.4% of the total 4,986 articles. Among the top publishing journals, *Pediatrics* leads with an IF of 8.0 and CPP of 89.71, while *Advances in Neonatal Care* has the lowest IF of 1.7 and CPP of 11.45. The average IF of the journals is 4.1 ± 2.42. According to the Science Citation Index Expanded (SCIE) of WoS, the journals fall into the categories *nursing*, *pediatrics*, *critical care medicine,* and *obstetrics and gynecology*.

**Table 2:** Top 10 publishing journals in the field of family-centered care.

Journal	Publication count	Citation per publication	Impact Factor (2022)
Pediatrics	138	89.71	8.0
Journal of Pediatric Nursing-Nursing Care of Children and Families	110	14.54	2.4
Journal of Clinical Nursing	102	20.40	4.2
Child Care Health and Development	93	20.72	1.9
Academic Pediatrics	70	30.13	3.1
Journal of Advanced Nursing	70	23.74	3.8
Journal of Obstetric Gynecologic and Neonatal Nursing	66	12.08	1.8
Critical Care Medicine	62	61.98	8.8
Intensive and Critical Care Nursing	54	12.67	5.3
Advances in Neonatal Care	53	11.45	1.7

### 3.4 Analysis of authors and publications

The 10 most frequently published authors are listed in Table 1. The highest publication count of 33 publications was recorded by Latour Jos M., while the highest CPP ratio of 57.55 was achieved by Curtis J. Randall. There were very few co-authorships among the top 10 publishing authors (Supplementary Figure S2). However, when considering the authors publishing at least 5 papers on FCC, all the top 10 authors except one are connected through co-authorships with other authors within a single network (Supplementary Figure S2).

Of the 4,986 FCC-related publications in the 21^st^ century, the most common publication types are articles (3,878; 77.8%), reviews (513; 10.3%), meeting abstracts (232; 4.7%) and editorial material (207; 4.2%) (Supplementary Table S3). The 10 most cited articles on FCC were cited on average 673.9 ± 184.54 times (Table 3). Most of them (6/10) are or comprise of guidelines or recommendations (2, 20–24) on how to best implement FCC. The others are focused on the perspectives, experience, and well-being of patients and loved ones (family, caregivers), also from the perspective of racially or ethnically diverse groups (25–28). The most cited article, with 1,082 citations, is entitled *Family Perspectives on End-of-Life Care at the Last Place of Care* by Teno et al. (25), published in 2004. The 10 articles with the highest yearly citation rate were cited with an average of 62.7 ± 19.99 citations/year. Among them were 4 of the 10 most cited papers: guidelines for the application of FCC on the Neonatal, Pediatric, and Adult ICU, the role of the pediatrician in FCC and family perspectives at end of life care (2, 22, 24, 25). The article with the highest yearly citation ratio of 119.2 is titled *Guidelines for Family-Centered Care in the Neonatal, Pediatric, and Adult ICU* by Davidson JE et al. (22). Among the articles with the highest yearly citation were also 3 related to COVID-19: managing the ICU surge (29), FCC applications in normal care (30) and telehealth opportunities (31).

**Table 3:** Top 10 most cited articles on total and yearly citation counts in the field of family-centered care.

Title	Author(s)	Publication year	Total citation count	Yearly citation count
Total most cited				
Family Perspectives on End-of-Life Care at the Last Place of Care	Teno JM et al.	2004	1,082	56.9
A consensus statement on health care transition for young adults with special health care needs	Blum R. *et al*	2002	808	38.6
Clinical practice guidelines for support of the family in the patient-centered intensive care unit: American College of Critical Care Medicine Task Force 2004–2005	Davidson JE et al.	2007	775	48.4
Guidelines for Family-Centered Care in the Neonatal, Pediatric, and Adult ICU	Davidson JE et al.	2017	715	119.2
The Health and Well-Being of Caregivers of Children With Cerebral Palsy	Raina P et al.	2005	686	38.1
Recommendations for end-of-life care in the intensive care unit: A consensus statement by the American College of Critical Care Medicine	Truog RD et al.	2008	675	45.0
Early Childhood Adversity, Toxic Stress, and the Role of the Pediatrician: Translating Developmental Science Into Lifelong Health.	Garner AS et al.	2012	579	52.6
Patient- and family-centered care and the pediatrician’s role	COMMITTEE ON HOSPITAL CARE and INSTITUTE FOR PATIENT- AND FAMILY-CENTERED CARE	2012	569	51.7
Current Research Findings on End-of-Life Decision Making Among Racially or Ethnically Diverse Groups	Kwak J, Haley W.E.	2005	434	24.1
A National Profile of the Health Care Experiences and Family Impact of Autism Spectrum Disorder Among Children in the United States, 2005–2006	Kogan MD et al.	2008	430	28.7
Highest yearly citation				
Guidelines for Family-Centered Care in the Neonatal, Pediatric, and Adult ICU	Davidson JE et al.	2017	715	119.2
Managing ICU surge during the COVID-19 crisis: rapid guidelines	Aziz S *et al*	2020	212	70.7
2022 Society of Critical Care Medicine Clinical Practice Guidelines on Prevention and Management of Pain, Agitation, Neuromuscular Blockade, and Delirium in Critically Ill Pediatric Patients With Consideration of the ICU Environment and Early Mobility	Smith HAB et al.	2022	66	66.0
Family-Centered Care During the COVID-19 Era	Hart JL et al.	2020	176	58.7
Family Perspectives on End-of-Life Care at the Last Place of Care	Teno JM et al.	2004	1,082	56.9
Early Childhood Adversity, Toxic Stress, and the Role of the Pediatrician: Translating Developmental Science Into Lifelong Health.	Garner AS et al.	2012	579	52.6
A Randomized Trial of a Family-Support Intervention in Intensive Care Units	White DB *et al*	2018	259	51.8
Patient- and family-centered care and the pediatrician’s role	COMMITTEE ON HOSPITAL CARE and INSTITUTE FOR PATIENT- AND FAMILY-CENTERED CARE	2012	569	51.7
Pediatric Teleheath: Opportunities Created by the COVID-19 and Suggestions to Sustain Its Use to Support Families of Children with Disabilities	Camden C; Silva M	2021	100	50.0
Clinical Practice Guideline for Screening and Management of High Blood Pressure in Children and Adolescents	Flynn JT et al.	2017	295	49.2

### 3.5 Analysis of keywords

**Figure 2:**
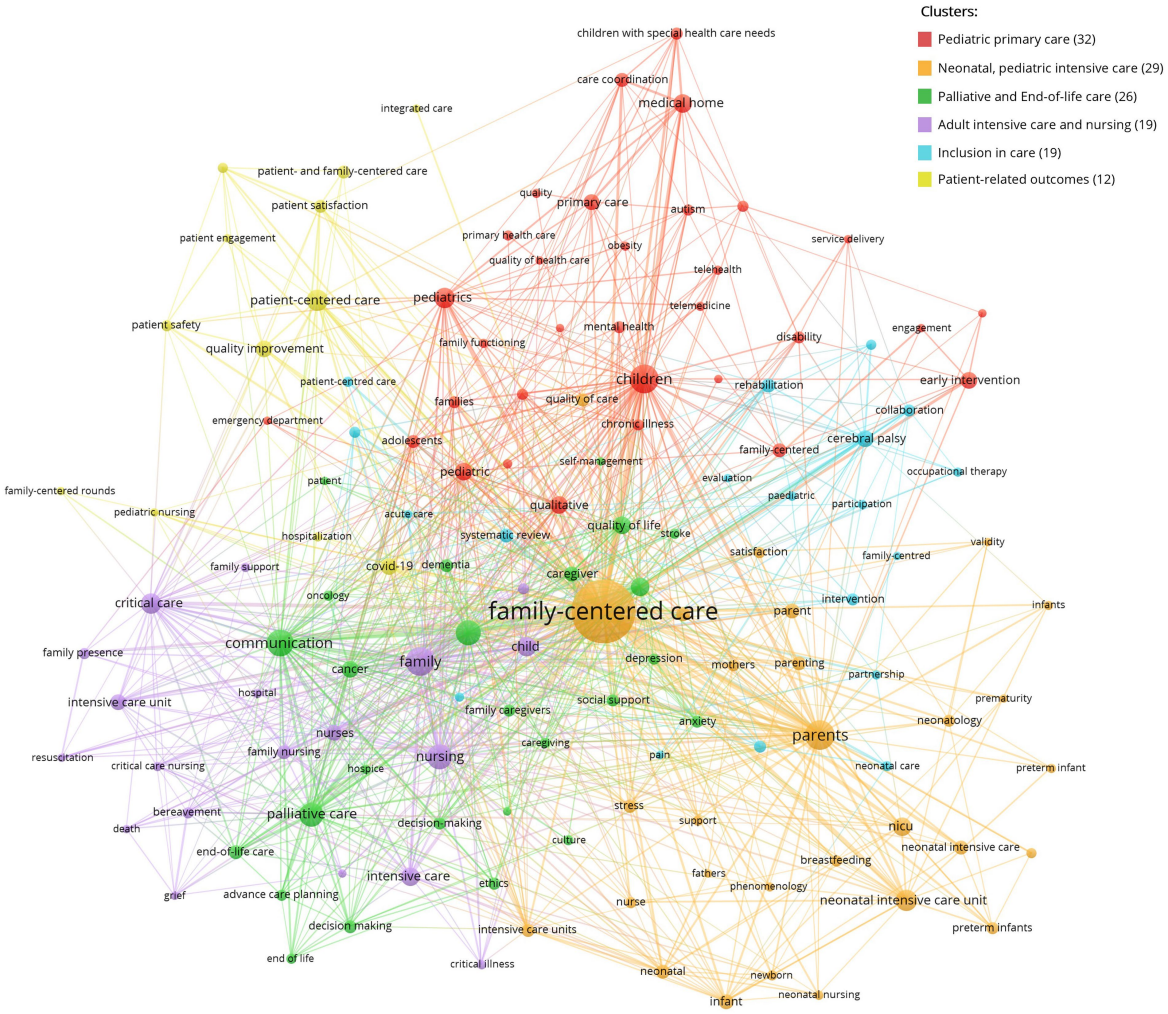
Top 2% authors keywords co-occurrence network of FCC publications in the 21st century.

Publications published in 2000 yielded 87 different author keywords, related to neonatology and intensive care, pediatrics and children with special care needs, parents and families (Supplementary Figure S4). The 4,986 FCC-related publications appearing between 2000 and 2022 yielded 7,272 unique author keywords, of which the top 140 (2%) are clustered and depicted in Figure 2. They form 5 different clusters: “pediatric and adolescent care,” “neonatology and pediatric intensive care,” “end-of-life care,” “adult intensive care and nursing,” “inclusion in care,” and “patient-related outcomes,” the main themes of FCC. In terms of publication year, among the earliest are “children with special care needs,” “medical home,” “chronic illness,” “neonatal nursing,” and “developmental care,” while the most recent are “Covid-19,” “telemedicine,” “telehealth” “patient,” and “patient engagement” (Supplementary Figure S5).

**Figure 3:**
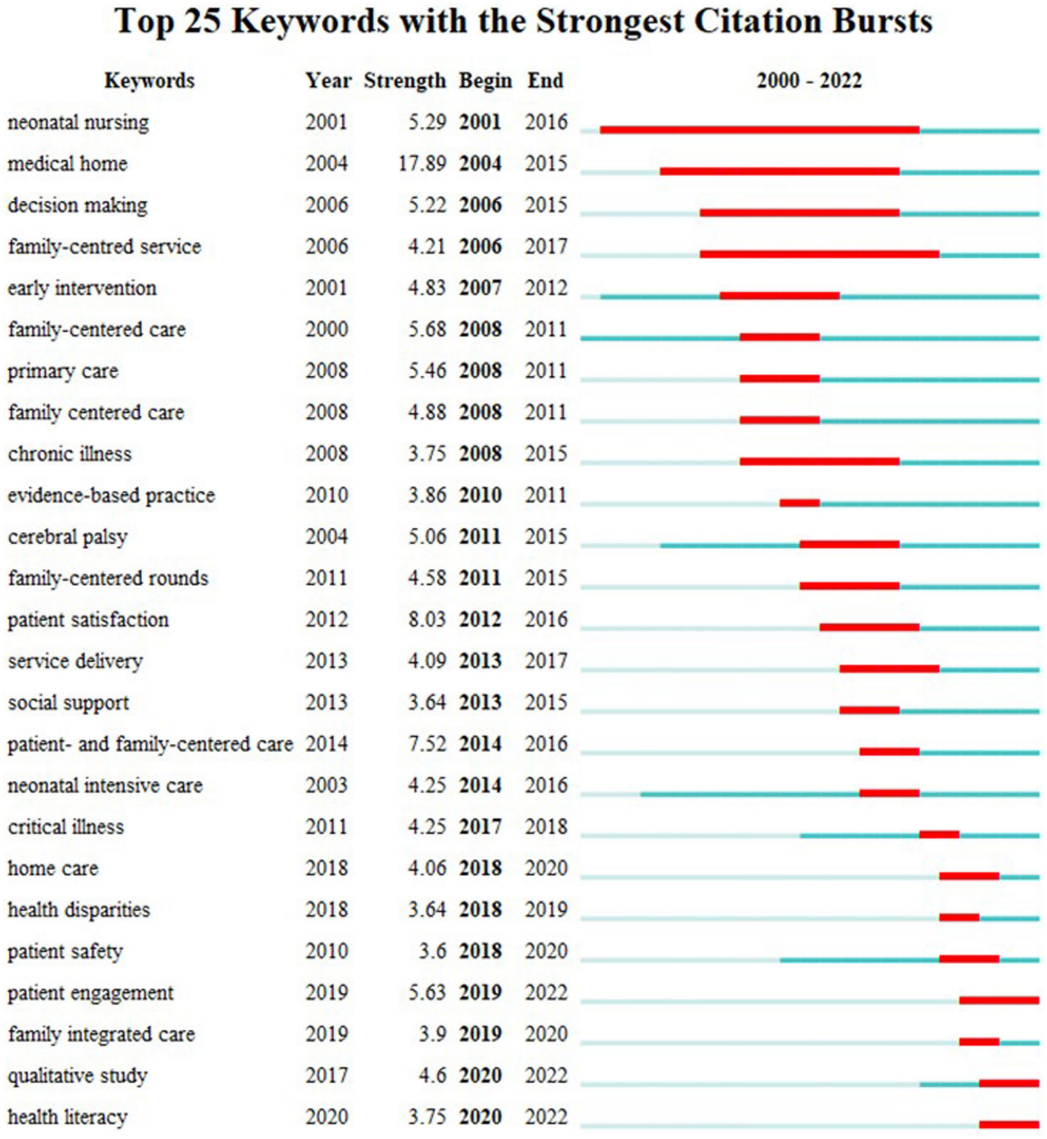
Top 25 author keywords experiencing citation bursts within the 21^st^ century in FCC publications. Blue represents the occurrence of the keyword while red represents the burst.

For author keywords, the top 25 experiencing citation bursts are shown in Figure 3. The earliest and longest lasting bursts were at the beginning of the 21^st^ century and are “neonatal nursing,” “medical home,” “decision making,” and “family-centered service,” with an average duration of 12.5 ± 2.18 years. The newest bursts lasting into 2022 are “patient engagement,” “qualitative study” and “health literacy,” lasting on average 3.3 ± 0.47 years.

## 4 Discussion

This bibliometric review shows a significant increase in publications on FCC in the 21^st^ century, spreading from the pediatric to the adult care setting, from a few to more than 100 countries worldwide, and with an increasing focus on patient experience and patient-reported outcomes. The largest areas of interest and application are nursing, neonatology and pediatrics, critical and intensive care, end-of-life (palliative) care and patient-related outcomes. The newest research areas are “health literacy,” “family involvement,” “family-integrated care” and “older adults.”

### 4.1 Publication output and growth in research interest

In the first decade of the 21^st^ century, FCC was still primarily associated with pediatric care (32). FCC experienced the strongest growth in publications since 2015, when “patient and family-centered care” (PFCC) experienced a burst in citations (Figure 2). This term was coined in the 1990s to give focus to engaging and viewing the patient and family/caregivers as an inseparable unit and essential members of the healthcare team and supporting them to increasing the patient’s health and quality of healthcare (3, 33). Although originating in pediatrics, this bibliometric review relates PFCC to many different applications across all healthcare settings.

There has been a recent paradigm shift in clinical research, especially with respect to clinical trials, with more active patient involvement and increased utilization of qualitative research methods focusing on patient-reported outcome measures (PROMs) and patient-reported experience measures (PREMs) (34–36). This can also be observed in our bibliometric review, as indicated by prominent clustering (Figure 2) for terms such as “patient satisfaction,” “patient-centered care,” “qualitative research,” “qualitative study,” and “patient experience.”

### 4.2 Leading countries, institutions and authors

FCC has developed from its activist origins in the pediatric sector in the United States and United Kingdom during the post-WWII era, when the experience of mass separations of children from their parents and children’s subsequent trauma were still very recent (32, 37). Furthermore, in some ways the term FCC reflects a response to western medical practice with its historically paternalistic system, in contrast to other cultures (often Asian and Hispanic) in which family involvement has always been the norm (28, 37). Overall, in terms of the number of published articles, top publishing authors and institutions, the leading proponents of FCC are the western English-speaking countries (United States, Canada, United Kingdom, and Australia). While there are international collaborations among researchers and organizations, most work on FCC is being conducted within tightly-knit working groups and national organizations (Figure 3) – a finding also described in a bibliometric review on palliative care research (13). While this allows for tailored approaches to the hospital and country settings, it may potentially result in institutional bias and a limited diversity of perspectives and potential exclusion of diverse viewpoints and experiences of different communities. Collaborations between institutions and countries are crucial for broad and inclusive application.

### 4.3 Current areas of application: beginning and end of life

Currently, FCC seems to be most relevant at the beginning of life, centering around neonatology and pediatrics, and at the end of life, centering around intensive, palliative and end-of-life care. In both stages, patients are more dependent on others, both physically and mentally. This is coupled with tightly embedded social and emotional family structures, forming an indivisible unit of patients and their family/caregivers that must be respected and utilized, but not disrupted, by medical care. However, as a consequence of medical progress in treating many chronic illnesses (e.g., diabetes mellitus, chronic kidney disease), as well as increasing life expectancy and prolongation of palliative care, statistically people spend more years in poor health and dependent on others (38–40). The recent increasing use of FCC in geriatric care is further indicated by the author keywords “dementia,” “family caregivers” and “quality-of-life” for very recent publication dates (Supplementary Figure S5). Willingness to care is negatively impacted by caregiver burden (41) however positively affected by reciprocal altruism and offering FCC approaches could further incentivize and support family caregiving.

### 4.4 Family-centered care and its forms of application

While sharing the principles of family or loved one integration, FCC has different characteristics when applied in different clinical settings.

The concept of family-centered care originates from pediatric primary care (Figure 2; Supplementary Figure S5), where FCC regards the patient, their parents and the family system as a single inseparable unit (2). However, the care differs according to the level of understanding and maturity of the patient, changing with the progression from infancy to adolescence and young adulthood, and with an increasing focus on the autonomy of the patient while still under parental guidance. One specific pediatric focus lies in children with severe chronic diseases, disabilities, and psychomotor retardation, as included in Figure 2 (disability, cerebral palsy, chronic illness). Here, the single inseparable unit of patient and family system and high level of received support does not significantly decrease as the child ages, reflecting the specific illness and degree of mental and physical maturity (42).

During the 21st century, FCC has also become closely associated with neonatal (intensive) care (Figure 2; Supplementary Figure S5). There, FCC not only ensure that infants are not separated from their parents, but also supports education of parents on relevant topics such as appropriate hygiene maintenance (43). COVID-19 related visitation restrictions showed the importance of FCC and the absence of mothers at the bedside lead to a cascade of disruptions to newborn care and breastfeeding practices (44). In recent years, the term family-integrated care has been gaining traction (Figure 3; Supplementary Figure S5). Family-integrated care focuses on the involvement family members in patient care to the extent that it allows families to perform care tasks that are normally performed by HCPs, to provide daily parent education programs (45), to enable parents to become primary caregivers in the neonatal intensive care unit (46) and to support lactation and breastfeeding (47).

In contrast, in the adult care setting the goal of FCC is to include a patient’s loved ones as equal partners in the provision of care, especially in the palliative and end-of-life setting (5).However usually adult healthcare is predominantly striving to be patient-centered with the aim to strengthen the patient-clinician relationship, promote communication about things that matter, help patients know more about their health, and facilitate their involvement in their own care (48). For patients who feel confident in managing their care independently, who have privacy concerns or strong personal values and beliefs profit from and may prioritize this approach. For example, a review of end-of-life decision making among racially or ethnically diverse groups shows not all ethnic groups are equally open to FCC (28). However health has a strong psychosocial aspect where social support, mental health, and emotional well-being have a high influence on a person’s well-being (49). Family-centered care recognizes the interconnectedness of family relationships and their impact on individual health outcomes and strives for the inclusion of the patients support system into the care plan. However patient privacy and autonomy are at the forefront of the needs to be guaranteed by the healthcare system. Therefore, FCC should be offered to the patient but introduced up to the level of the patients wishes. Then, effective communication between healthcare providers, patients, and their families is crucial to understanding and respecting the specific wishes and needs of each patient but also their family members.

### 4.5 Integration into adult healthcare

It could be argued that FCC is less relevant during adulthood compared to the beginning and end of life, because, unless suffering from chronic illness involving countless diagnostic and therapeutic interventions with frequent follow-up visits and in-patient stays, for most adults medical visits or temporary medical conditions are infrequent occurrences. However, humans are social creatures and there is an extraordinary amount of interdependence in adults, not just dependency of children on adults. It is often independent of how serious the medical problems or preventive or clinical care that happens is that patients seek support and try to contextualize the problem within their socioecological system. In a recent study conducted by our group, we found a wish for adult health care to be generally more like pediatrics (6), in the sense of considering the patient as a whole including its social support circle.

As seen in Figure 2, FCC associates with SDM, which is relevant in pediatric and adult care settings (50), as well as in advance care planning, a process in which individuals make decisions about the health care they wish to receive when they are no longer able to make their own medical decisions (51) and which often includes family members in the process (40). Involving family members in adult medical care in general should be considered not only in cases where the patient has a legally appointed representative or surrogate decision maker, but also for routine medical care. Furthermore, by involving families in the care process, healthcare providers can gain valuable insights and perspectives that can contribute to safer and more effective care (3). However, in many cases, a collaborative approach that combines elements of both person-centered and family-centered care may be the most effective in meeting the diverse needs of patients.

### 4.6 Family-centered care and patient safety

FCC is closely related to patient safety, as shown in Figures 2, 3. Improved communication, coordination, and engagement of patients and their loved ones allows healthcare plans to be better communicated and risks better identified and mitigated (3) and patient participation is advocated as a means to improve patient safety (52). Furthermore, it increases psychological safety especially of patients and loved ones, which in turn supports patient satisfaction and improves the patient experience. In the United States, FCC has been officially recognized as an intervention that promotes safety and quality of care, and hospitals in the United States are actively encouraged to incorporate patient- and family-centered practices (2). Such initiatives should also be adopted in other countries.

### 4.7 Family-centered care in the age of digitalization

As well as the constant need for social support and having loved ones present throughout the healthcare journey, interest in FCC might also be influenced by digitalization and our culture of information sharing, as well as generational changes in the behavior and value systems of patients and health care providers (53–55). Our modern information age is probably also a contributing factor in the growing interest in FCC. Furthermore, the Internet gives patients and their loved ones greater access to information, leading to increased health literacy, but also greater misinformation and misconceptions about diagnoses, diagnostics, and treatment options (56). Therefore, it is even more critical that medical information is communicated in a trustworthy and reliable manner, irrespective of whether by an institution or a single healthcare provider. Increasing health literacy is one of the main strategies for promoting PFCC (33), and the “health literacy” citation burst reflects this development (Figure 3).

With respect to the modern information age, two of the author keywords associated with FCC are “telemedicine” and “telehealth” (Figure 2). Although telemedicine solutions were already in existence prior to the pandemic (57–59), their expansion was facilitated by COVID-19 (60, 61). However, while telemedicine and telehealth have positive outcomes and enjoy acceptance among HCPs, patients, and their loved ones, the solutions have not yet been widely adopted.

### 4.8 Limitations

To remain as current as possible, our literature search strategy included all complete years in the 21^st^ century. However, as Web of Science is continuously and retrospectively updated, and as there can be delays in indexing by some journals, the bibliometric data for the last couple of years may be subject to minor changes. While more literature databases are available, the data for this study was only extracted from the Web of Science Core Collection, which is standard practice for most bibliometric analyses (62). This however has the drawback that it does not index all literature on a topic, but only selected articles and journals as described by Clarivate (63). In order to capture all published articles on FCC, we did not limit the article type. A limitation of the VOS Viewer is that all authors of a publication are included in the analysis. Therefore, according to the affiliations of its authors, a publication can be attributed to several countries and organizations. Lastly, while there are many different indices for measuring the impact of publications, we only used the total count and citations of author/organization/country/article impact, which are generally the most known and frequently used metrics.

## 5 Conclusion

Scientific interest in FCC is growing and during the first 22 years of the 21^st^ century has spread from the pediatric to the adult intensive, palliative and end-of-life settings. It is closely connected with patient safety, experience and satisfaction. The recent research directions include involving patients in the development of FCC strategies, health literacy interventions, and the uptake of telemedicine solutions.

## Data availability statement

The original contributions presented in the study are included in the article/supplementary material, further inquiries can be directed to the corresponding author.

## Author contributions

MH: Conceptualization, Data curation, Formal analysis, Investigation, Methodology, Software, Visualization, Writing – original draft, Writing – review & editing. FE: Conceptualization, Formal analysis, Validation, Visualization, Writing – review & editing. NB: Formal analysis, Writing – review & editing. AY: Methodology, Software, Writing – review & editing. AA: Writing – review & editing, Data curation. ES: Conceptualization, Supervision, Writing – review & editing.
