# Supplementary material for: Research areas and trends in family-centered care in the 21st century: a bibliometric review
Source: Front Med (Lausanne). 2024 Jun 11;11:1401577. doi: 10.3389/fmed.2024.1401577 (PMC11201138; doi:10.3389/fmed.2024.1401577)

**Supplementary Figure S1: Network of top 2% of publishing institutions on FCC.
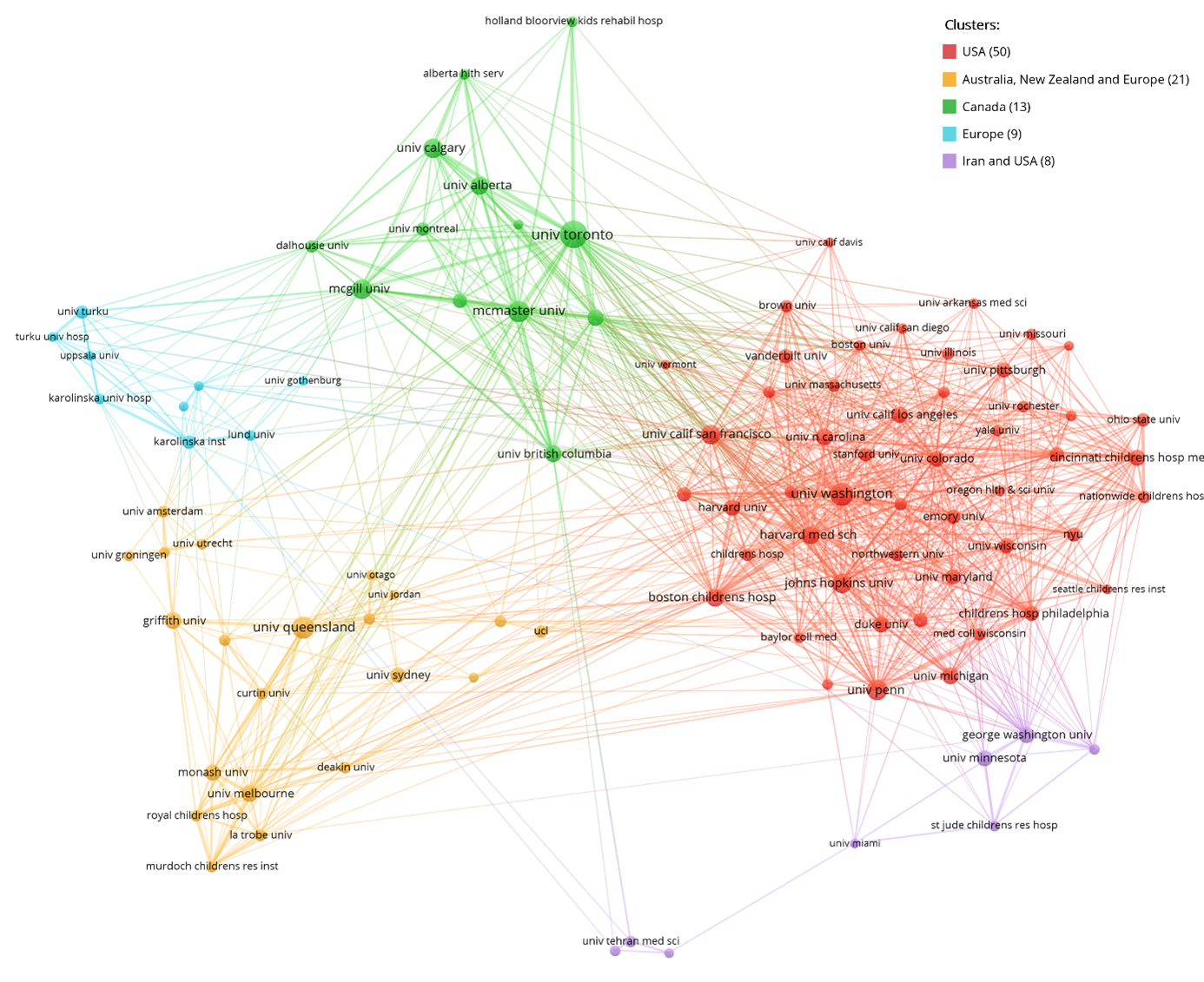
**

**Supplementary Figure S2: Cooperation network of authors within FCC.** Authors publishing 5 or more publications were considered. Each color represents a different network of co-authoring authors.

**
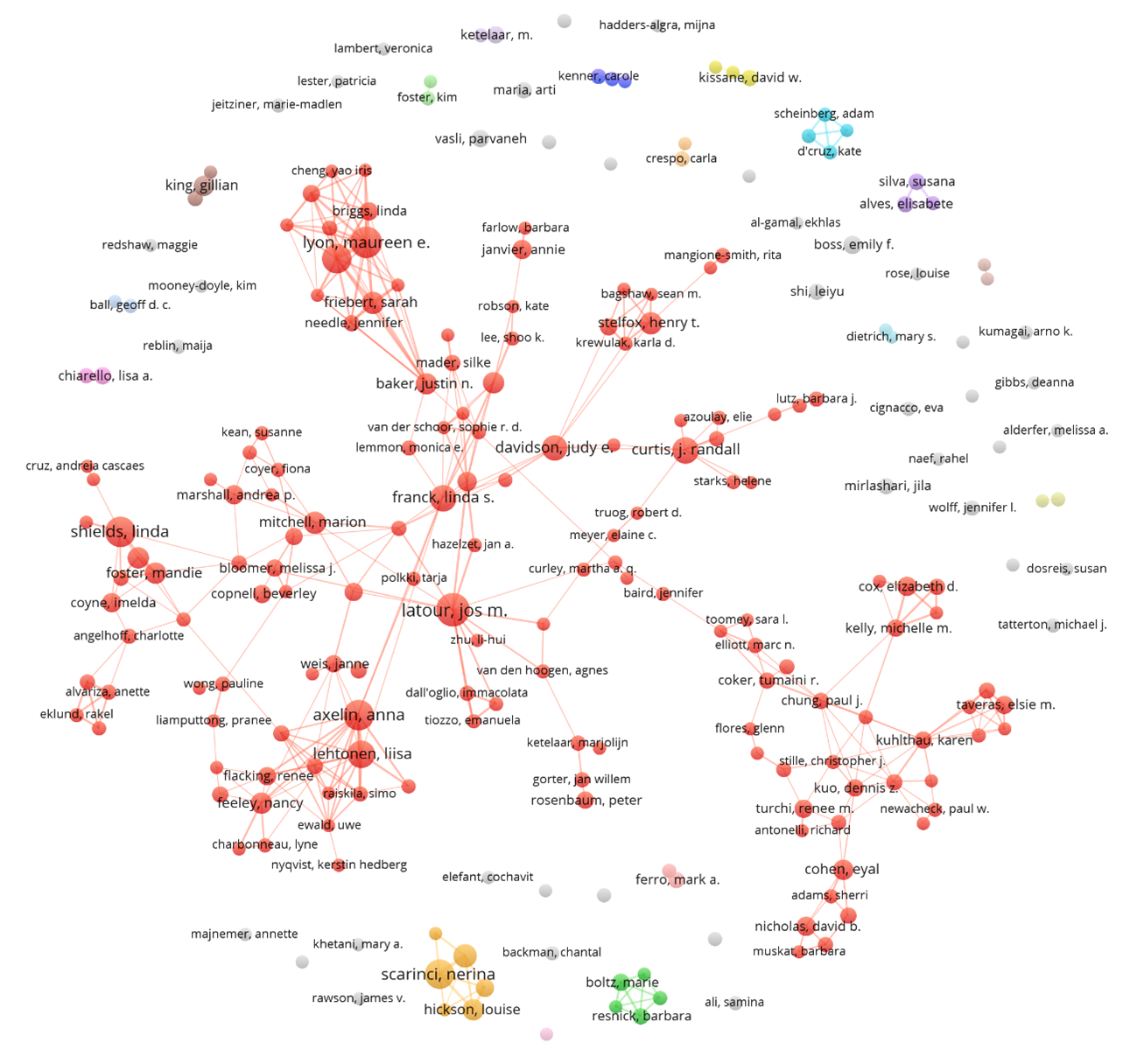
**

**Supplementary Table S3: Publication types included in the bibliometric review**

| **Publication Type** | **Number of publications** |
| --- | --- |
| Article | 3878 |
| Review Article | 513 |
| Meeting abstract | 232 |
| Editorial material | 207 |
| Proceeding paper | 38 |
| Early access article | 33 |
| Letter | 24 |
| Book review | 12 |
| Others (correction, book chapter, news item, reprint, retracted publication) | 23 |

**Supplementary Figure S4: Authors keywords co-occurrence network of FCC publications in the year 2000.** All keywords are shown.


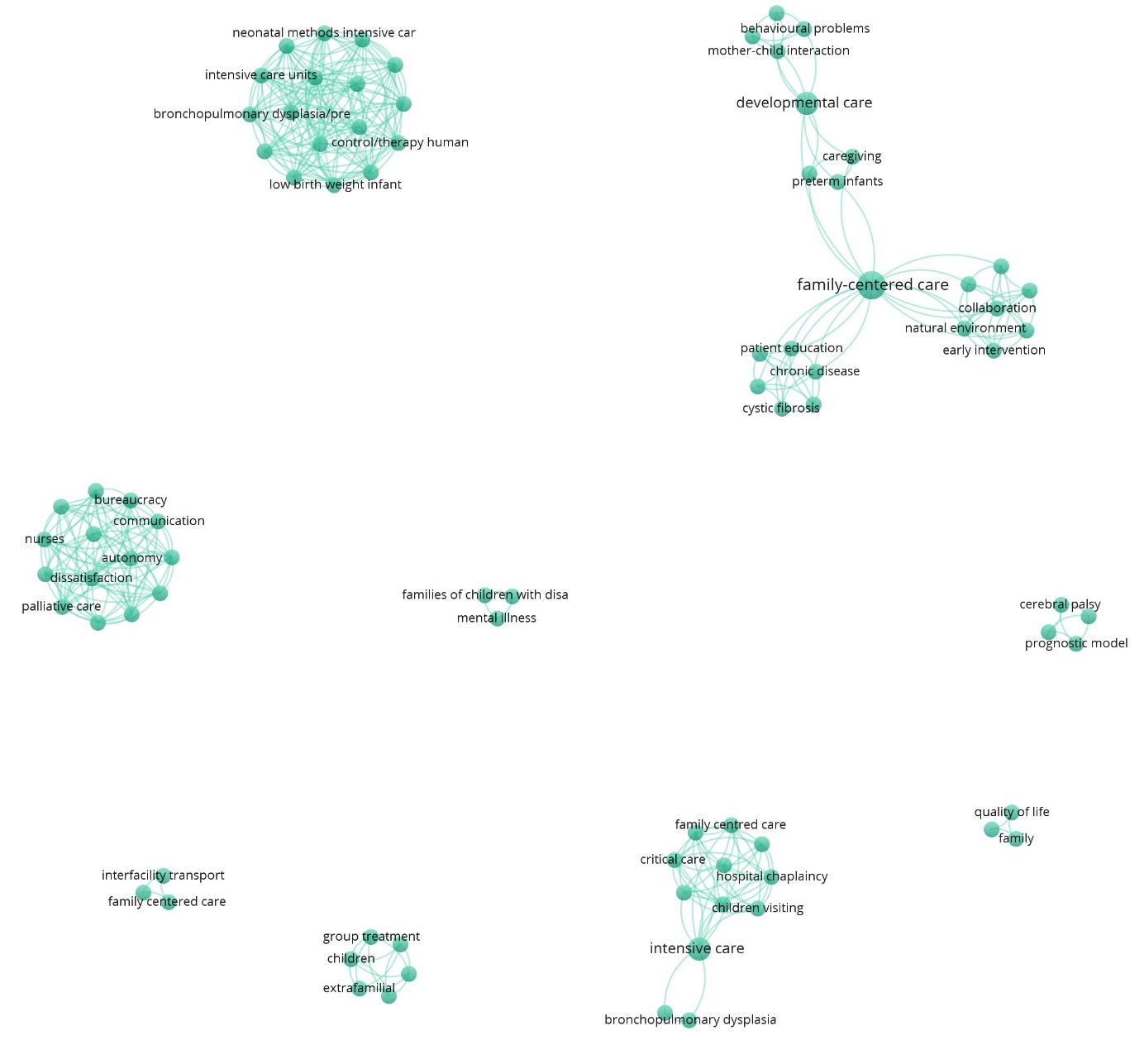


**Supplementary Figure S5: Average publication dates of authors keywords in co-occurrence network of FCC publications in the 21^st^ century.**


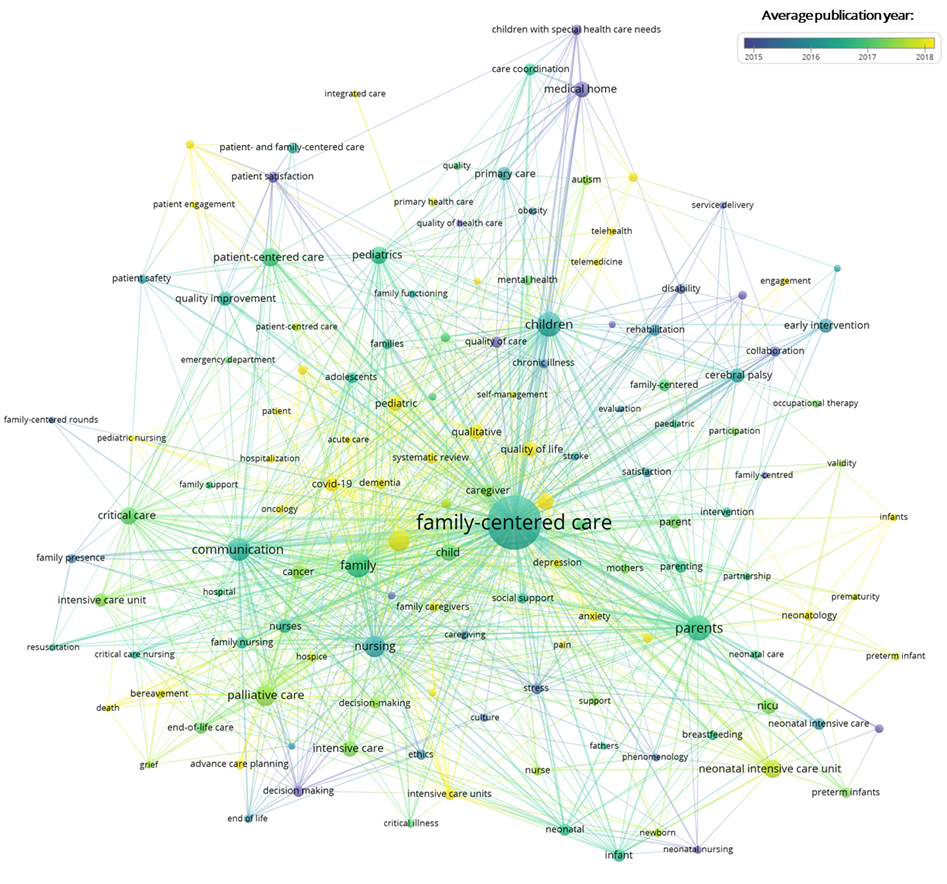

Supplement: Supplementary file 1 [file Data_Sheet_1.docx]
